# Early predictors of new-onset immune-related seizures: a preliminary study

**DOI:** 10.1186/s12883-022-03042-0

**Published:** 2022-12-29

**Authors:** Xiangsong Shi, Weiwei Cai, Xiulin Zhang, Heyue Pan, Chengbing Huang, Shouyong Wang, Jianyang Xu

**Affiliations:** 1 Department of Neurology, Huai’an Third People’s Hospital, No. 272, Huaihai West Road, 223001 Huai’an, China; 2 Department of Psychiatry, Huai’an Third People’s Hospital, 223001 Huai’an, China

**Keywords:** Seizures, Immune-related, MRI, Neuronal autoantibodies, APE2

## Abstract

**Background:**

Approximately 60% of patients with autoimmune encephalitis (AE) exhibit secondary acute symptomatic seizures and showed highly sensitive to immunotherapy. However, it is difficult for many patients to receive early immunotherapy since the early identification of the cause in AE is more complex. This study aimed to investigate the early predictors of initial immune-related seizures and to guide the evaluation of treatment and prognosis.

**Methods:**

One hundred and fifty-four patients with new-onset “unknown etiology” seizures with a course of disease less than 6 months were included. Serum and/or cerebrospinal fluid neuron-specific autoantibodies (NSAbs), including N-methyl-D-aspartate receptor (NMDAR), α-amino-3-hydroxy-5- Methyl-4-isoxazole propionic acid receptor 1 (AMPAR1), AMPAR2, anti-leucine rich glioma inactivated 1 antibody (LGI1), anti-gamma-aminobutyric acid type B receptor (GABABR), anti-contact protein-related protein-2 (CASPR2) were used to screen for immune etiology of the seizures. In addition, patients with epilepsy and encephalopathy were also examined via brain MRI, long-term video EEG, antibody prevalence in epilepsy and encephalopathy (APE2) score, and modified Rankin Scale (mRS). A logistic regression model was used to analyze the early predictors of immune etiology.

**Results:**

Thirty-four cases (22.1%) were positive for NSAbs. Among all 154 patients, 23 cases of autoimmune encephalitis (AE) (21 cases of NSAbs positive), 1 case of ganglionic glioma (NSAbs positive), 130 cases of epilepsy or seizures (12 cases of NSAbs positive) were recorded. Also, there were 17 patients (11.0%) with APE2 ≥ 4 points, and all of them met the clinical diagnosis of AE. The sensitivity and specificity of APE2 ≥ 4 points for predicting AE were 73.9% and 100%. The results of multivariate analysis showed that the NSAbs and APE2 scores independently influenced the early prediction of initial immune-related seizures (*P* < 0.05).

**Conclusion:**

NSAbs and APE2 scores could act as early predictors of initial immune-related seizures.

## Background

Epilepsy is one of the most common neurological diseases, with a lifetime prevalence of 7.60/1000 (95% CI 6.17–9.38) and an incidence of 61.44/100,000 years (95% CI 50.75–74.38), affecting approximately 50 million people worldwide [1]. It is characterized by a persistent tendency to seizures with neurological, cognitive, psychological, and social impairment [2]. Epilepsy severely impacts the quality of life of the patient and has an economic burden of approximately 0.5% of the global disease burden [3]. Acute symptomatic seizures occur with systemic injury or have a close temporal association with documented brain injury, with an incidence of 29–39/100,000 person-years. Differ from epilepsy, the etiology of acute symptomatic seizures is often clear, such as stroke, traumatic brain injury, etc., and these seizures have a low rate of recurrence [4]. Early identification of the cause of seizures clinically is considered to be critical for the early detection and management of associated causes and for implementing interventions to reduce the majority of preventable seizures.

With the recent advancements in neuroimmunology, especially after the International League Against Epilepsy (ILAE) listed “immunity” as one of the six major causes of epilepsy in 2017 [5], autoimmune encephalitis (AE) has garnered a lot of attention. Common clinical manifestations of AE include seizures, cognitive impairment, psychobehavioral abnormalities, movement disturbances, autonomic dysfunction, and disturbance of consciousness [6, 7]. In addition, approximately 60% of patients with AE exhibit secondary acute symptomatic seizures. Early immunotherapy has been shown to be associated with good clinical outcomes [8, 9]. However, it is difficult for many patients to achieve an early identification and immunotherapy since the clinical manifestations of AE are complex, and the early identification of the cause is more complex than stroke and traumatic brain injury. Currently, the diagnosis of AE relies on the detection of neuron-specific autoantibodies (NSAbs). However, due to the popularity of antibody testing, time cost, and interpretation of test results, the challenge of early identifying the cause of immune-related seizures in clinical practice is substantial. This study aimed to investigate the early predictors of initial immune-related seizures and to guide the evaluation of treatment and prognosis.

## Materials and methods

### Research subjects

Between August 2016 and December 2021, 154 patients with initial “unknown etiology” seizures diagnosed and treated by the Epilepsy Center of the Third People’s Hospital of Huai’an City,Jiangsu Province were enrolled. Inclusion criteria: course of disease ≤ 6 months, seizures can’t be explained as a known epilepsy etiology, risk factors for epilepsy can be excluded, such as perinatal injury, focal neurological impairment, family history of epilepsy.The classification of seizures was consistent with the 2017 ILAE’s new classification of seizures and epilepsy [5]. The diagnosis of epilepsy was based on the 2014 ILAE pragmatic clinical definition of epilepsy [10]. Among the patients, there were 89 males and 65 females, and the median age was 23 (2–84). Clinical manifestations: 154 cases of epileptic seizures, including 14 patients with abnormal mental behavior, 8 subjects with cognitive impairment, 5 patients with facial-brachial dystonic seizures (FBDS), and 1 case with other facial movement disorders.

### Methods

**Fig. 1:**
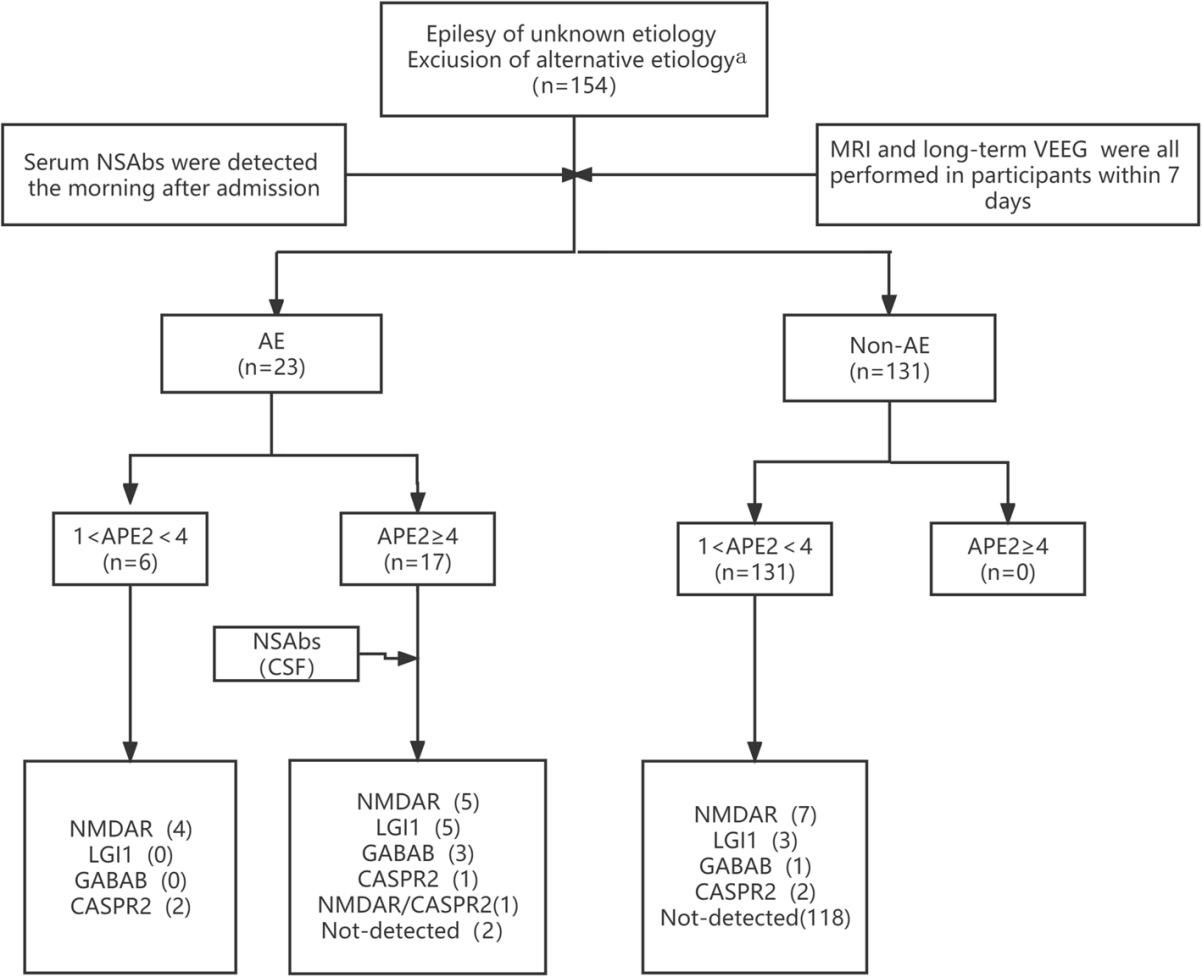
Autoimmune epilepsy diagnostic criteria stratified as per Antibody Prevalence in Epilepsy and Encephalopathy (APE2) score and neural antibody status. ^a^Reasonable exclusion of alternative etiology (genetic, infectious encephalitis, neoplasm, neurodegenerative process, or metabolic or toxic encephalopathy)

The Study is a prospective cohort study. There were 23 subjects in the AE cohort (mean age: 48.74 ± 20.36 years; male/female: 12/11) and 131 subjects in the Non-AE cohort (mean age: 26.79 ± 20.43 years; male/female: 53/78) included in our cohort study.As shown in Fig. 1, for the participants, peripheral venous blood was drawn in the morning the day after admission. Antibodies were detected using a cell-based assay (CBA, KingMed Diagnosis®), each one containing transfected cells expressing the receptors of a different neuronal surface antigen:NMDAR, LGI1, CASPR2, GABABR, AMPA1R, and AMPA2R. Further, six NSAbs were detected in cerebrospinal fluid (CSF) of patients with an APE2 score ≥ 4 within 7 days.

In EPILEPSIA, Dubey et al. reported that the APE 2 score, a 10-item, 18-point scale, could be used to investigate risk factors for seropositivity of neural antibodies predictive of an autoimmune cause for epilepsy [11]. According to Husari’s study, we took APE2 score ≥ 4 as a meaningful criterion [12].

Demographic characteristics, clinical data, biological findings were collected (Table 1). Brain magnetic resonance imaging (MRI) and long-term video EEG were all performed in participants within 7 days.

**Table 1:** Summarizes the participants’ demographic and clinical characteristics

	AE	Non-AE	*P* values
Sample size	23(14.9%)	131(85.1%)	
Age of onset(year)			<0.01
<16	0/23(0%)	58/131(36.7%)	
≥ 16	23/23(100%)	73/131(47.4%)	
Sex			0.294
Male	12/23(52.2%)	53/131(40.4%)	
Female	11/23(47.8%)	78/131(59.6%)	
APE2			<0.01
<4	6/23(26.1%)	131/131(100%)	
≥ 4	17/23(73.9%)	0/131(0%)	
NSAbs			
Total	21	13	<0.01
NMDAR	9/21(42.9%)	7/13(53.8%)	
GABABR	3/21(14.3%)	1/13(15.4%)	
LGI1	5/21(23.8%)	3/13(23.1%)	
CASPR2	3/21(14.3%)	2/13(7.7%)	
NMDAR/CASPR2	1/21(4.7%)	0/13(0%)	

Then we performed related checks to screen for oncologic comorbidities such as ovarian teratoma, small-cell lung cancer or thymoma.

### Statistical analysis

A logistic regression model was used for multivariate analysis. The diagnosis of immune-related seizures was used as the dependent variable. The Independent variables included indicators with statistical significance in the above analysis and were included in the logistic regression equation for multivariate analysis. *P* < 0.05 was considered to be statistically significant.

## Results

### Positive rate of NSAbs

As shown in Table 2, among 154 patients, 34 (22.1%) were found to be positive for NSAbs. Of the 6 neuronal autoantibodies screened, 16/34 (47.1%) NMDAR, 8/34 (23.5%) LGI1, 5/34 (14.7%)CASPR2, 4/34 (11.8%)GABABR, 1/34 (2.9%) were positive for CASPR2 and NMDAR double antibodies, while AMPA1R or AMPA2R antibody positive cases were not detected. Based on the AE diagnostic criteria [6], among 34 NSAbs-positive patients, 21 were diagnosed with AE, all aged ≥ 16 years old, 9/21 (42.9%) were NMDAR positive, 5/21 (23.8%) LGI1 positive, 3/21 (14.3%) GABABR positive, 3/21 (14.3%) CASPR2 positive, 1/21 (4.7%) CASPR2 and NMDAR double-antibody positive. Of 120 NSAbs negative cases, only 2 NSAbs-negative patients were diagnosed with antibody-negative AEs. The sensitivity and specificity of NSAbs positive index in diagnosing AE were 91.3% and 90.1%. patients diagnosed with AE were administered immunotherapy or/and anti-seizure medications (ASMs). Among them, 22 cases were in remission, and 1 case died of lung cancer. ASMs alone in non-AE patients achieved remission in 125 cases and ineffectiveness or deterioration in 6 cases.

**Table 2:** Clinical and laboratory characteristics of epilepsy patients with diagnosed AE and NSAbs (+)

Age	Sex	NSAbs	APE2 Score	Clinical diagnosis of AE	MRI limbic system	Epilepsy risk factors	Video-EEG epileptiform discharge	Concomitant tumor	ASM	Immunity therapy	mRS	
											On admission	Six months later
21	Female	NMDAR	8	Yes	Yes	No	No	Ovarian teratoma	1	IVMP, IVIg, PL EX,Pred	4	0
66	Male	Not detected	7	Yes	No	No	No	No	2	IVMP, IVIg	3	1
63	Male	LGI1	7	Yes	Yes	No	No	No	0	IVMP,Pred	3	1
62	Male	LGI1	7	Yes	No	No	No	No	2	IVDXM	3	1
74	Female	LGI1	6	Yes	No	No	No	No	2	IVMP,IVIg,Pred	3	1
71	Male	GABABR	6	Yes	Yes	No	No	Lung cancer	1	IVMP,Pred	4	6
17	Female	NMDAR	6	Yes	No	No	No	Ovarian teratoma	1	IVMP,IVIg,PLEX,Pred	4	1
16	Female	NMDAR	6	Yes	No	No	No	Ovarian teratoma	1	IVMP,IVIg,Pred	3	0
71	Male	LGI1	5	Yes	Yes	No	Yes	No	1	IVMP	3	1
58	Male	NMDAR	5	Yes	No	No	No	No	0	IVMP,Pred	3	1
39	Female	CASPR2	5	Yes	No	No	No	Thymoma	1	IVMP,IVIg, Pred	3	0
34	Female	GABABR	5	Yes	Yes	No	No	No	1	IVMP,IVIg,Pred	4	0
55	Female	GABABR	4	Yes	Yes	No	No	No	1	IVMP,Pred	3	0
49	Female	LGI1	4	Yes	No	No	No	No	0	IVMP,Pred	4	0
34	Male	Not detected	4	Yes	No	No	No	No	1	IVMP,IVIg, Pred	3	0
25	Female	NMDAR	4	Yes	No	No	No	Ovarian teratoma	1	IVMP,IVIg,Pred	3	0
17	Female	CASPR2/NMDAR	4	Yes	No	No	Yes	No	0	IVDXM	3	0
74	Male	CASPR2	3	Yes	No	No	No	No	1	IVMP,IVIg, Pred	3	0
73	Male	NMDAR	3	Yes	No	No	No	No	0	IVMP	3	0
72	Female	NMDAR	3	No	No	No	Yes	No	1	None	1	1
21	Female	LGI1	3	No	Yes	No	Yes	Ganglioglioma	1	None	1	1
20	Male	CASPR2	3	No	No	No	Yes	No	2	None	1	1
62	Male	NMDAR	2	Yes	No	No	No	No	1	IVMP,Pred	4	3
55	Female	NMDAR	2	Yes	No	No	No	No	1	IVMP,IVIg, Pred	4	2
53	Female	NMDAR	2	Yes	No	No	No	No	0	IVMP,Pred	3	0
32	Male	CASPR2	2	Yes	No	No	No	No	1	IVDXM	2	1
72	Male	LGI1	1	No	No	No	Yes	No	1	None	1	0
68	Male	LGI1	1	No	No	No	Yes	No	1	None	1	1
52	Male	NMDAR	1	No	No	No	Yes	No	0	None	1	0
36	Male	NMDAR	1	No	No	No	No	No	1	None	1	0
17	Female	CASPR2	1	No	No	No	No	No	1	None	1	1
14	Male	NMDAR	1	No	No	No	Yes	No	1	None	1	0
10	Female	NMDAR	1	No	No	No	Yes	No	1	None	1	0
10	Male	NMDAR	1	No	No	No	No	No	1	None	1	1
8	Female	GABABR	1	No	No	No	Yes	No	1	None	1	0
2	Female	NMDAR	1	No	No	No	Yes	No	0	None	1	1

*IVMP* Intravenous methylprednisolone, *IVIg* Intravenous immunoglobulin, *IVDXM* Intravenous dexamethasone, *PLEX* Plasma exchange, *Pred* Prednison

### APE2 score

The median APE2 score for all patients was 1 (1–8). There were 17 patients (11.0%) with APE2 ≥ 4 points, and all of them met the clinical diagnosis of AE. There were 137 patients (89.0%) with a score of 1 ≤ APE2 < 4, including 6/137 (4.4%) in the AE group and 131/137 (95.6%) in the non-AE group (Table 1). The sensitivity and specificity of APE2 ≥ 4 for the diagnosis of AE were 73.9% and 100%, respectively.

### Brain MRI

All patients underwent brain MRI examination via the Shanghai United Imaging MR 1.5T operation. The primary sequences included cross-sectional T1WI, T2WI, DWI, and fluid-attenuated inversion recovery (FLAIR) sequences, with a scanning slice thickness of 5 mm. Unilateral or bilateral abnormal signals in the medial temporal lobe WERE was observed in 12 patients, including eight unilateral and four bilateral; six patients in the AE group and six in the non-AE group.

**Fig. 2:**
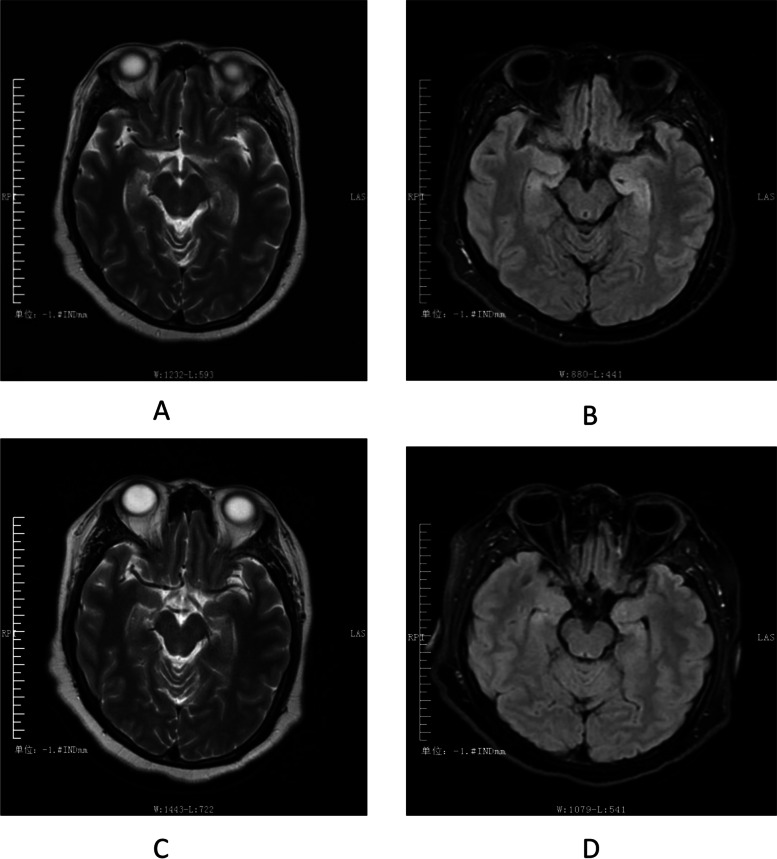
MRI image of the patient (Example 1). T2W1 showed a high signal (**a**), Flair showed a high signal (**b**), and the abnormal signal in the hippocampus disappeared after six months of immunotherapy (**c**, **d**)

**Fig. 3:**
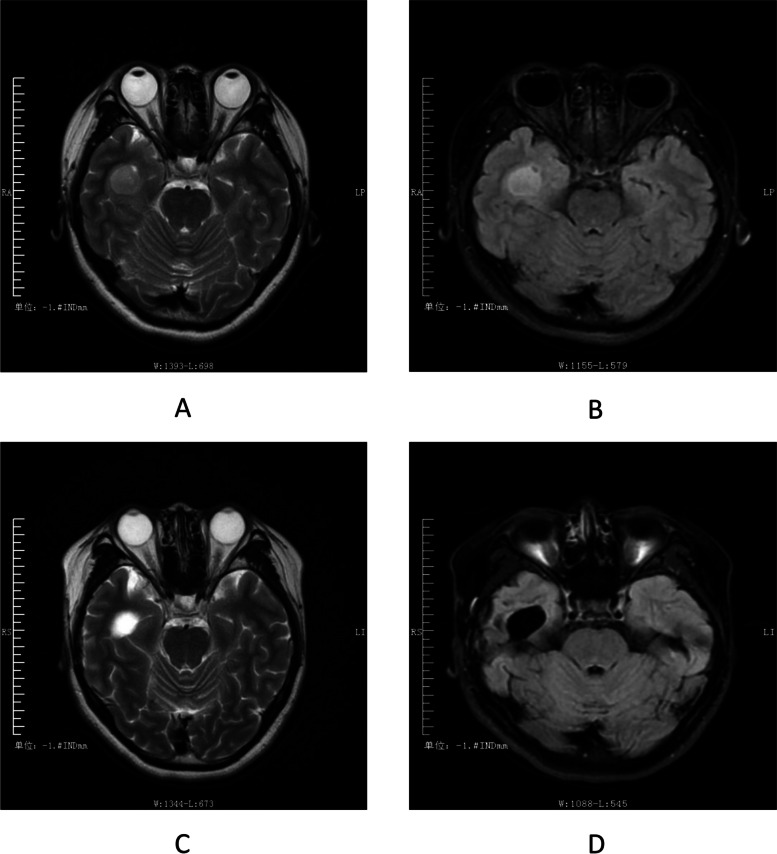
MRI image of the patient (Example 2). T2W1 was a slightly high signal (**a**), and Flair was a high signal (**b**). Postoperative re-examination showed that T2W1 was a high signal (**c**) and Flair was a low signal (**d**)

Example 1: A 34-year-old female with a diagnosis of GABABR encephalitis. The brain MRI scan showed that the bilateral hippocampus was swelling, with a high signal in T2W1 and a high signal in Flair. such abnormal signals in the hippocampus completely disappeared after Six months of immunotherapy (Fig. 2). Example 2: A 21-year-old female was diagnosed with ganglioglioma and positive serum LG1. The brain MRI scan showed a round mass of about 2.0*1.6 cm in the right temporal lobe, with a slightly high signal on T2W1 and a high signal on Flair. Postoperative follow-up showed that T2W1 was hyperintensity and Flair was hyperintensity (Fig. 3).

### Long-range video EEG

All patients underwent long-range video EEG monitoring using Nihon Kohden 1100-K long-range EEG monitoring system (64 channels). Interictal epileptiform discharges (IEDs) were observed in 120 patients, including five patients in the AE group. Sixteen cases showed focal slow wave activity or rhythm, among them ten subjects were AE. 18 patients showed no abnormality, among them 8 subjects were AE.

### Concomitant tumor

There were 4 cases of NMDAR(+) with ovarian teratoma, 1 case of GABABR(+) with lung cancer, and 1 case of CASPR2(+) with thymoma. The serum antibody titer of patients with tumor was higher than that of patients without tumor.

### Multivariate analysis of early predictors of initial immune-related seizures

The results showed that NSAbs and APE2 scores were independent factors for the early prediction of initial immune-related seizures (*P* < 0.05, Table 3).

**Table 3:** Multivariate analysis of early predictors of initial immune-related seizures

Variables	β value	SE value	Wald χ^2^ value	*P* value	OR value (95%CI)
Gender	-0.931	1.297	0.515	0.473	0.394 (0.031 ~ 5.007)
Age (years)	0.037	0.031	1.403	0.236	1.038 (0.976 ~ 1.103)
NSAbs positive	4.07	1.581	6.623	0.01	58.555 (2.639 ~ 1299.231)
APE2	2.189	0.737	8.83	0.003	8.929 (2.107 ~ 37.835)
MRI abnormalities	-0.421	1.729	0.059	0.807	0.656 (0.022 ~ 19.445)

Variable assignment: gender, male = 1, female = 2; age, actual value; NSAbs positive, no = 0, yes = 1; APE2, actual value; abnormal brain MRI, no = 0, yes = 1

## Discussion

There has been an immense improvement in our understanding of immune-related seizures with the development of NSAbs detection technology in recent years. This study aimed to investigate the early predictors of immune-related seizures based on clinical features and neuroimaging, EEG, and NSAbs.

NSAbs, as biomarkers for the early identification of AEs, are valuable for immune-related seizures [13]. Several studies have shown that the positive incidence of NSAbs in the serum of epilepsy patients without genetic, structural, or metabolic etiologies is 15–20% [14–16]. Currently, in the diagnostic criteria of AE [7], three months is the time limit for subacute onset. However, recent studies have shown that the onset of antibody-mediated seizures is occasionally indeterminate, even weeks or months before the diagnosis of an AE. Therefore, it is difficult to define an operational time definition [17] clinically. In this observational study, we found NSAbs-positive serum or cerebrospinal fluid prevalence in 22.1% of 154 patients with initial “unknown etiology” seizures with a duration of ≤ 6 months. These results suggest that even in patients with epileptic seizures of “unknown etiology” with a period longer than three months, testing for NSAbs is still clinically significant and might increase the recognition of immune-associated epileptic seizures.

A prospective clinical study found that neuronal antibody positivity in adults with new-onset epilepsy was not supportive of an immune etiology [18]. Here, 12/34 (35.2%) of NSAbs-positive patients had only a single seizure without other clinical features of AE. After treatment with ASM alone, the patients exhibited a good prognosis, and no new AE symptoms were observed during long-term follow-up, which was not consistent with the diagnosis of AE. This result showed that in patients with epileptic seizures with initial “unknown etiology,“ NSAbs positivity could not be used as the only basis for immune etiology and needs to be combined with the clinical characteristics of patients, which was consistent with the results of the Zelano et al. [18].

FBDS is a characteristic manifestation of LGI1 encephalitis. The literature has reported that FDG-PET in patients with FBDS often indicates metabolic abnormalities in the basal ganglia, indicating that FBDS may be subcortical epilepsy [19, 20]. In this group of LGI1 patients, 5/6 (83.3%) developed FBDS at different stages of the disease. One patient had frequent attacks of FBDS in the early stage of the disease. Combination therapy of the two ASMs was reported to be ineffective. During the ictal and interictal periods, EEG showed no epileptiform discharges, blood and cerebrospinal fluid NSAbs were negative, and the APE2 score was 7. Post clinical diagnosis of antibody-negative AE and simultaneous pulse therapy with methylprednisolone, the seizures gradually reduced and eventually disappeared.The APE2 score made up for the insufficiency of NSAbs detection. However, we also found it sometimes difficult to distinguish viral encephalitis from AEs using the APE2 score. NSAbs test results could be used as a good supplement, but the second-generation sequencing technology for viral etiology is not very popular in China. Therefore, diagnosing antibody-negative immune-related seizures must be done carefully to avoid magnification of the diagnosis.

Among the patients diagnosed with immune-related seizures, each subtype of NSAbs was 42.9% for NMDAR, 23.8% for LGI1, 14.3% for GABABR, 14.3% for CASPR2, and 4.7% for CASPR2 and NMDAR double-antibody positive, consistent with previous studies [15]. Also, the sensitivity of NSAbs detection in serum and cerebrospinal fluid samples of patients with different subtypes was variable [21, 22]. The positivity rate of cerebrospinal fluid in NMDAR subtypes was higher than that in serum, while LGI1 and CASPR2 were the opposite; for patients with combined tumors, antibodies were more easily detected in serum. We found that low serum titers (1:10) of NMDAR were insignificant in patients with seizures alone, and serum antibody titers were significantly higher in patients with tumors than those without tumors. It is suggested that detecting NSAbs in cerebrospinal fluid and serum is the best choice in patients with suspected immune-associated epileptic seizures. However, due to patient cooperation and informed consent, cerebrospinal fluid testing is sometimes difficult to implement.

APE2 is a predictive model established based on clinical assessment and related auxiliary examination results and can be easily operated in clinical practice. Husari et al. proposed that NSAbs should be detected in all epilepsy patients with unknown etiology and APE2 score ≥ 4 [12]. This study showed that the APE2 score ≥ 4 accounted for approximately 73.9% of patients with AE. Currently, the clinical application of domestic antibody detection is limited by time and technology. APE2 could be used as an effective supplement to antibody detection in early clinical evaluation, especially patients with “unknown etiology” epilepsy underwent antibody detection before or without antibody detection conditions. Also, patients with APE2 scores ≥ 4 should be tested for NSAbs in cerebrospinal fluid. Better sensitivity and specificity results also indicated that for patients with APE2 score ≥ 4, NASbs detection in CSF and serum could be performed simultaneously; for patients with APE2 score < 4, serum detection alone could be considered.

In this study, we also found that “immune” and “structural” etiologies could occur simultaneously in a patient. Brain MRI is one of the etiological examinations for initial epilepsy or encephalitis, especially the abnormal signal of T2/FLAIR in one or both medial temporal lobes could indicate AE [23]. Our study showed that 6/23 (26.1%) patients in the AE group had abnormal neuroimaging findings, supporting the findings of previous studies [24]. Although the results of multivariate analysis showed that brain MRI could not be used as an independent indicator for early prediction (*P* = 0.807), the abnormal brain MRI lesions in a few patients with AE could be reversibly changed with immunotherapy (Fig. 2). In addition, a right temporal lobe space-occupying lesion was found on the brain MRI of one LG1-positive non-AE patient (Fig. 3), and the postoperative pathological diagnosis was ganglioglioma. Therefore, a brain MRI is necessary to evaluate the early etiology and prognosis of epileptic seizures. Epilepsy surgeons should fully consider immune etiologies in the preoperative evaluation of patients with temporal lobe structural disease [25].

The results of long-term video EEG showed that IEDs accounted for 77.9% of all patients. However, the detection rate of IEDs in patients with immune-associated epileptic seizures was not high, of which about 43.4% (10/23) showed focal slow wave activity or rhythm. However, some studies showed that IEDs might be a risk factor for the recurrence of epilepsy in patients with all types of AE [26].

Also, multivariate analysis showed that both NSAbs and APE2 were independent factors for the early prediction of immune-related seizures (*P* < 0.05). This further highlighted the importance of NSAbs and APE2 in the diagnosis and treatment of this disease. Guidelines including “A clinical approach to diagnosis of autoimmune encephalitis” [7] and “Expert Consensus on Diagnosis and Treatment of Autoimmune Encephalitis in China” [27] emphasize the importance of NSAbs; however, there needs to be greater awareness regarding the importance of APE2 score. Interestingly, it has been recently proposed that the diagnosis of AE should not be confined to positive antibody, but should be guided by clinical features [28]. Our study supports this conclusion and more attention needs to be paid to the early diagnosis and prognostic assessment of immune-related seizures.

This study also had certain limitations: First, lack of longitudinal follow-up of antibody-positive cases. Second, although the results showed good statistical power, the number of samples was smaller than that of similar studies. In future studies, longitudinal long-term follow-up needs to be conducted based on the observation of a larger sample size, which would result in a comprehensive and in-depth analysis of the significance of NSAbs and APE2 scores in the diagnosis and treatment of immune-related seizures.

## Conclusion

Our study found that immune-related seizures were more common with positive NSAbs and APE2 score ≥ 4, and neuroimaging facilitated the diagnosis of the disease. We demonstrated that NSAbs and APE2 scores could be used as early predictors of initial immune-related seizures. APE2 score combined with NSAbs detection could help early identification of immune etiologies while avoiding the expansion of detection scope and immunotherapy. Additionally, early immunotherapy was found to be beneficial for the prognosis of immune-mediated seizures. Patients with single seizures who were NSAbs-positive might not need immunotherapy. Our results provide a testable hypothesis for future RCTs study.

## Data Availability

The datasets generated and/or analysed during the current study are not publicly available because further publications are still being analysed from the data. However, data are available from the corresponding author on reasonable request.

## Abbreviations

AE: Autoimmune encephalitis
AMPAR: α-amino-3-hydroxy-5- Methyl-4-isoxazole propionic acid receptor
APE2: Antibody prevalence in Epilepsy and encephalopathy score
ASMs: Anti-seizure medications
CASPR2: Anti-contact protein-related protein-2
CBA: Cell-based assay
FBDS: Facial-brachial dystonic seizures
FLAIR: Fluid-attenuated inversion recovery
GABABR: Anti-gamma-aminobutyric acid type B receptor
IEDs: Interictal epileptiform discharges
ILAE: International League Against Epilepsy
LGI1: Anti-leucine rich glioma inactivated 1 antibody
mRS: Modified Rankin Scale
NMDAR: N-methyl-D-aspartate receptor
NSAbs: Neuron-specific autoantibodies
